# Predictors of mother and child DNA yields in buccal cell samples collected in pediatric cancer epidemiologic studies: a report from the Children’s Oncology group

**DOI:** 10.1186/1471-2156-14-69

**Published:** 2013-08-12

**Authors:** Jenny N Poynter, Julie A Ross, Anthony J Hooten, Erica Langer, Crystal Blommer, Logan G Spector

**Affiliations:** 1 Division of Epidemiology and Clinical Research, Department of Pediatrics, University of Minnesota, Minneapolis, MN, USA; 2 Masonic Cancer Center, University of Minnesota, Minneapolis, MN, USA; 3 Department of Pediatrics, MMC 715, 420 Delaware St. S.E., Minneapolis, MN 55455, USA

**Keywords:** DNA collection, Buccal cells, Pediatric epidemiology

## Abstract

**Background:**

Collection of high-quality DNA is essential for molecular epidemiology studies. Methods have been evaluated for optimal DNA collection in studies of adults; however, DNA collection in young children poses additional challenges. Here, we have evaluated predictors of DNA quantity in buccal cells collected for population-based studies of infant leukemia (N = 489 mothers and 392 children) and hepatoblastoma (HB; N = 446 mothers and 412 children) conducted through the Children’s Oncology Group. DNA samples were collected by mail using mouthwash (for mothers and some children) and buccal brush (for children) collection kits and quantified using quantitative real-time PCR. Multivariable linear regression models were used to identify predictors of DNA yield.

**Results:**

Median DNA yield was higher for mothers in both studies compared with their children (14 μg vs. <1 μg). Significant predictors of DNA yield in children included case–control status (β = −0.69, 50% reduction, *P* = 0.01 for case vs. control children), brush collection type, and season of sample collection. Demographic factors were not strong predictors of DNA yield in mothers or children in this analysis.

**Conclusions:**

The association with seasonality suggests that conditions during transport may influence DNA yield. The low yields observed in most children in these studies highlight the importance of developing alternative methods for DNA collection in younger age groups.

## Background

Biological samples are frequently collected in epidemiologic studies to evaluate genetic susceptibility to disease. Blood samples provide a large quantity of high quality DNA; however, collection of blood is invasive, expensive and typically not feasible in studies covering a large geographic area. Importantly, the use of non-invasive DNA collection methods has been shown to increase study participation rates [1]. Buccal and saliva samples are a non-invasive and inexpensive way to collect DNA in population-based studies, which, in turn, has been successfully evaluated using high-throughput genotyping methods [2-4].

Genetic variants are especially likely to be relevant in pediatric diseases such as cancer due to the early age of onset;[5,6] however, collection of DNA from infants and young children poses significant challenges [7-11]. While blood samples resulted in higher DNA yields, [7,9] data suggest that buccal cell samples from children also provide sufficient DNA for genotyping for a limited number of loci [7,9-11]. Studies comparing different methods of buccal cell collection have indicated that treated cards, specifically FTA Micro cards, may result in better genotyping results than buccal swab samples, although other complexities, including difficulty processing the samples, were noted [10,11]. These studies have also suggested that whole genome amplification (WGA) can be successfully used to increase DNA yields in buccal cells collected from children [7,11]. In adult studies, mouthwash collection of buccal cells and collection of saliva samples using commercial kits have shown increased DNA yields [12-15]; however, these methods are not feasible for young children.

In this analysis, we evaluated predictors of DNA quantity in mothers and children from buccal samples collected for population-based studies of infant leukemia and hepatoblastoma (HB) conducted through the Children’s Oncology Group (COG).

## Results

### Characteristics of the study population

DNA samples were available for 427 children and 462 mothers in the HB study, and 396 children and 494 mothers in the infant leukemia study. Mothers and children with missing maternal interview data were excluded from the analyses (N = 15 HB children and mothers, 4 infant leukemia children and 5 infant leukemia mothers). Selected characteristics of children and mothers with DNA and interview data are shown in Table 1.

**Table 1:** Selected characteristics of the study population

***Characteristic***	***HB study***	***Infant leukemia study***^***a***^
	***N (%)***	***N (%)***
N children with samples	412	392
Median DNA yield (μg)	0.37 (0 – 139)	0.15 (0 – 14)
Child’s age at DNA collection		
0 – 2 years	45 (11)	75 (19)
2 – 5 years	151 (37)	123 (31)
> 5 years	216 (52)	194 (49)
Child’s sex		
Male	241 (58)	183 (47)
Female	171 (42)	209 (53)
Case control status		
Case	246 (60)	223 (57)
Control	166 (40)	169 (43)
Storage (Child’s sample)		
0 – 3 days	108 (29)	121 (46)
3 – 7 days	99 (26)	64 (24)
7 – 14 days	92 (24)	54 (21)
> 14 days	79 (21)	23 (9)
Season of DNA collection		
Spring	119 (29)	117 (30)
Summer	117 (28)	80 (20)
Fall	90 (22)	109 (28)
Winter	86 (21)	86 (22)
Sample collection		
Cytosoft brush	175 (42)	341 (87)
Epicentre brush	202 (49)	51 (13)
Mouthwash	35 (9)	
N Mothers with samples	446	489
Median DNA yield (μg)	14 (0.02 – 401)	14 (0.06 – 1209)
Mother’s age at DNA collection		
< 30 years	89 (20)	104 (21)
30 – 35 years	117 (26)	147 (30)
35 – 40 years	131 (29)	134 (27)
> 40 years	109 (24)	104 (21)
Storage (Mother’s sample)^a^		
0 – 3 days	110 (27)	121 (38)
3 – 7 days	126 (31)	104 (33)
7 – 14 days	100 (25)	57 (18)
> 14 days	68 (17)	35 (11)
Maternal education		
< High School	31 (7)	26 (5)
High School	74 (17)	104 (21)
Some college	115 (26)	146 (30)
College degree	158 (35)	152 (31)
Advanced degree	68 (15)	61 (13)
Maternal race		
White	349 (78)	411 (84)
Black	18 (4)	19 (4)
Hispanic	50 (11)	30 (6)
Asian	18 (4)	16 (3)
Other	11 (2)	13 (3)
Income		
< $20,000	65 (15)	73 (15)
$20,000 -- $50,000	122 (28)	183 (38)
$50,000 -- $75,000	102 (23)	110 (23)
>%75,000	153 (35)	122 (25)
Season of DNA collection		
Spring	128 (29)	138 (28)
Summer	126 (28)	100 (20)
Fall	98 (22)	140 (29)
Winter	94 (21)	111 (23)

^a^ N’s may not sum to total due to missing data. Storage time missing for 34 children in the HB study, 130 children in infant leukemia study, 42 mothers in HB study, and 172 mothers in the infant leukemia study. Income data were missing for 4 mothers in the HB study and 1 mother in the infant leukemia study.

Approximately half of the children in both studies were under age 5 years at the time of DNA collection. Approximately 60% of the DNA samples were from cases in both studies. DNA extraction was initiated within one week of receipt of DNA for the majority of samples. There was no correlation between DNA yield in mother and child pairs (r = −0.006, *P* = 0.86). As expected, DNA yield was significantly higher in mothers than children (t Value two-sample *t*-test with unequal variance = 34.85, p < 0.0001). The yield was much higher for the 35 children in the HB study who were old enough to provide DNA by mouthwash collection (median 6.5 μg, range 0—139 μg) compared to the children who provided DNA by buccal brush collection (median 0.26 μg, range 0—12 μg).

### Predictors of DNA yield in children

Results from multivariable linear regression models evaluating predictors of DNA yield are shown in Table 2. Models were adjusted for all covariates included in the table. Duration of storage in the laboratory prior to DNA extraction and maternal education were also evaluated; however, these variables were not confounders and were not included in the final models. In children from the HB study, significantly lower DNA yields were obtained for cases than controls, children whose mother reported race other than white, and for samples received during the summer months. In addition, samples collected using the Epicentre brush or mouthwash yielded significantly higher amounts of DNA than samples collected with the Cytosoft brush. Similar results for case control status, brush type and season of DNA collection were observed in the infant leukemia study, although the results for case–control status did not reach statistical significance. In the analysis of children from both study populations, significant associations were observed for case–control status, sample collection type, and season of DNA collection. DNA yields were significantly higher for children in the HB study than for children in the infant leukemia study. Because of the large difference in DNA yield by sample collection type, we repeated the analysis after excluding the 35 children in the HB study who provided DNA through mouthwash collection. The results of the analysis were unchanged when these individuals were removed.

**Table 2:** Predictors of DNA Yield (log_e transformed) in children

	***HB (N = 412)***			***Infant Leukemia (N = 392)***			***Combined (N = 804)***		
	**β**^**a **^**(95% CI)**	**% change**^**b**^	***P *value**	**β**^**a**^	**% change**^**b**^	***P *value**	**β**^**a**^	**% change**^**b**^	***P *value**
Age at DNA collection (years)									
0 – 2	0.60 (−0.27-1.47)	81	0.18	0.81 (−0.37-1.99)	125	0.18	0.67 (−0.09-1.42)	95	0.08
2 – 5	−0.10 (−0.65-0.46)	−9.1	0.74	−0.76 (−1.72-0.20)	−53	0.12	−0.42 (−0.97-0.12)	−34	0.13
> 5 years	Reference	Reference		Reference	Reference		Reference	Reference	
Child’s sex									
Male	Reference	Reference		Reference	Reference		Reference	Reference	
Female	0.06 (−0.42-0.55)	6.6	0.80	0.47 (−0.35-1.28)	60	0.26	0.31 (−0.17-0.78)	36	0.21
Study									
HB	NA	NA	NA	NA	NA	NA	Reference	Reference	
Infant leukemia	NA	NA	NA	NA	NA	NA	−1.05 (−1.60- -0.51)	−65	0.0002
Case control status									
Control	Reference	Reference		Reference	Reference		Reference	Reference	
Case	−0.65 (−1.18- -0.11)	−48	0.01	−0.64 (−1.52-0.25)	−42	0.16	−0.69 (−1.20- -0.17)	−50	0.01
Collection type									
Cytosoft brush	Reference	Reference		Reference	Reference		Reference	Reference	
Epicentre brush	0.94 (0.43-1.45)	160	0.0003	2.46 (1.15-3.76)	1070	0.0002	1.23 (0.64-1.81)	242	<0.0001
Mouthwash	4.22 (3.26-5.17)	6670	<0.0001	NA		NA	4.42 (3.16-5.67)	8210	<0.0001
Maternal race									
White	Reference	Reference		Reference	Reference		Reference	Reference	
Other	−0.66 (−1.24- -0.08)	−49	0.03	0.36 (−0.75-1.47)	43	0.52	−0.19 (−0.80-0.42)	−17	0.52
Season of DNA collection									
Spring	Reference	Reference		Reference	Reference		Reference	Reference	
Summer	−0.89 (−1.52- -0.26)	−59	0.006	−3.85 (−5.03- -2.66)	−97	<0.0001	−2.06 (−2.71- -1.42)	−87	<0.0001
Fall	−0.36 (−1.05-0.33)	−30	0.31	−1.56 (−2.67- -0.45)	−79	0.006	−0.84 (−1.50- -0.19)	−57	0.01
Winter	0.01 (−0.67-0.70)	1.5	0.97	1.02 (−0.13-2.17)	469	0.08	0.54 (−0.13-1.22)	72	0.11

^a^ Models were adjusted for all other variables in table.

^b^ Percent change in DNA yield compared to referent.

We also evaluated season of DNA collection separately for each DNA collection method. In a combined analysis of all children, we found a significant difference for seasonality of DNA collection for samples collected with the Cyto-Pak Cytosoft brushes (β = −2.95, *P* < 0.0001 for summer vs. spring) but not for samples collected with the Epicentre Catch-All Sample Collection swabs (β = −0.80, p = 0.09 for summer vs. spring) or for the mouthwash samples (β = −0.33, *P* = 0.63 for summer vs. spring).

### Predictors of DNA yield in mothers

No significant predictors of DNA yield were identified in mothers in the HB study (Table 3). In mothers from the infant leukemia study, associations were observed for the mother’s age at DNA collection, case control status of her child, and season of DNA collection. In the combined model, mothers over age 40 years at DNA collection had significantly lower DNA yield than mothers younger than 30. Samples received in the fall and winter had higher yields than samples received in the spring. This is in contrast to the findings in the children, where yields were significantly lower in samples collected in the fall and there was no significant difference between samples collected in the spring and winter (Table 2).

**Table 3:** Predictors of DNA Yield (log_e transformed) in mothers

	***HB (N = 446)***			***Infant leukemia (N = 489)***			***Combined (N = 935)***		
	**β**^**a**^	**% change**^**b**^	***P *value**	**β**^**a**^	**% change**^**b**^	***P *value**	**β**^**a**^	**% change**^**b**^	***P *value**
Age at DNA collection (years)									
< 30 years	Reference	Reference		Reference	Reference		Reference	Reference	
30 – 35 years	0.06 (−0.35-0.47)	6.2	0.78	−0.25 (−0.67-0.16)	−22	0.23	−0.11 (−0.41-0.18)	−10	0.45
35 – 40 years	−0.16 (−0.56-0.24)	−15	0.43	0.04 (−0.39-0.46)	4.1	0.86	−0.09 (−0.38-0.21)	−8.6	0.57
> 40 years	−0.21 (−0.62-0.21)	−19	0.33	−0.47 (−0.93- -0.01)	−37	0.04	−0.37 (−0.68- -0.05)	−31	0.02
Study									
HB	NA	NA	NA	NA	NA	NA	Reference	Reference	
Infant leukemia	NA	NA	NA	NA	NA	NA	0.05 (−0.15-0.26)	5.1	0.63
Case control status									
Control	Reference	Reference		Reference	Reference		Reference	Reference	
Case	−0.13 (−0.41-0.16)	−12	0.38	0.41 (0.09-0.72)	51	0.01	0.14 (−0.08-0.36)	15	0.21
Maternal race									
White	Reference	Reference		Reference	Reference		Reference	Reference	
Other	0.10 (−0.23-0.44)	11	0.54	0.13 (−0.27-0.53)	14	0.53	0.13 (−0.14-0.39)	14	0.34
Season of DNA collection									
Spring	Reference	Reference		Reference	Reference		Reference	Reference	
Summer	0 (−0.37-0.37)	0	1.00	0.30 (−0.12-0.73)	35	0.16	0.18 (−0.10-0.46)	20	0.20
Fall	−0.19 (−0.57-0.20)	−18	0.35	0.82 (0.43-1.21)	127	<0.0001	0.39 (0.11-0.66)	48	0.007
Winter	0.25 (−0.15-0.65)	28	0.22	0.46 (0.04-0.87)	58	0.03	0.35 (0.06-0.64)	42	0.02

^a^ Models were adjusted for all other variables in table.

^b^ Percent change in DNA yield relative to referent.

## Discussion

In this analysis of DNA yield from buccal cells collected in two population-based case–control studies of childhood cancer, we identified several predictors of DNA yield including case–control status of the child and season of DNA collection. The samples collected from cases in both studies had a significantly lower DNA yield compared with samples from controls. The child samples also had a lower yield if they were collected in the summer months compared with the spring, which was consistent across both study populations.

Studies in adults have reported higher DNA yields from mouthwash samples compared with buccal brush samples [12,16-18]. In this analysis, we also found that this method yielded higher amounts of DNA in children who were old enough to provide a mouthwash sample. Proposed explanations for the higher yield associated with mouthwash collection include inhibition of bacterial growth in the sample during transport and storage due to the alcohol content of the mouthwash [16] and reduced DNA degradation in the mouthwash samples during collection and mailing at room temperature [12]. This well-documented increased yield indicates that this method may be ideal for collecting DNA in older children; however, this method is not suitable for use in very small children.

We observed a significantly lower DNA yield in samples collected from children during the summer months. This difference was observed only in the child samples, and the magnitude of this difference was larger in the infant leukemia study compared with the HB study. This finding can be attributed to differences in DNA collection by age group and study as this finding was limited to the Cyto-Pak Cytosoft brushes, which were used only in children and in a higher proportion of children in the infant leukemia study. To our knowledge, this effect of seasonality has not been reported previously; one potential explanation is DNA degradation caused by increased bacterial growth at higher temperatures. In a recent study of DNA collected for a study of infants, Gallagher et al. [19] reported higher DNA yield and quality in cytobrush samples that were allowed to air dry compared with samples collected by standard collection methods (i.e. storage in a plastic tube) presumably due to reduced bacterial growth in samples not stored in humid conditions. If confirmed in additional studies, a drying procedure may increase feasibility in pediatric populations where DNA collection is difficult.

In our study, DNA samples from cases yielded a significantly lower quantity of DNA compared with samples from controls. This finding is in contradiction to an Australian study of children with acute lymphoblastic leukemia where median DNA yield was higher in 31 cases compared to 52 control children [10]. However, DNA collection was completed in a clinic setting for a proportion of the cases, where participant adherence to instructions could be monitored, so this could at least partially explain this discrepant finding. The explanation for the lower yield of DNA in cases compared with controls is not obvious. One potential explanation is the possibility that mothers used less vigorous swabbing in the cases due to oral sensitivity following chemotherapy. Alternatively, technical variation (i.e. a hidden batch effect) confounded by case–control status could also explain the finding.

Demographic variables within the mother and child subgroups were not strong predictors of DNA yield. Within the samples collected by buccal brush, there were no differences in DNA yield by age group. In previous studies, DNA yield was higher for males than females [20,21]; however, we did not observe such a difference in either pediatric population. Previous studies have reported lower DNA yield in individuals of non-white race [21]. While we observed a lower DNA yield in the HB study for children where the mother reported non-white race, no difference was observed in the infant leukemia study nor in the mothers in either study. As the majority of both study populations was white, we had limited power to detect racial differences in DNA yield.

This study measured DNA yield; however, the real parameter of interest is the ability to successfully conduct genotyping assays on the resulting DNA. SNP genotyping at a limited number of loci has been conducted in samples from both of the study populations. In a recent study evaluating four SNPs in the infant leukemia study, genotyping was successful for 171/189 (90%) samples included [22]. Similarly, the majority of the cases in the HB study were also successfully genotyped using the Sequenom platform (343/386 with complete triad data, 88% success rate; Spector el al. unpublished data). For more extensive genotyping applications, such as GWAS, the number of cases with sufficient DNA is more limited. For example, only 31% of cases in the HB study and 21% of cases in the infant leukemia study have a total DNA yield greater than or equal to 1 μg.

There are a number of strengths associated with this evaluation, including the population based data collection for both children and adults. In addition, DNA quantity was measured using qPCR and therefore provides an accurate measure of human DNA quantity. The availability of systematically measured covariates is also a strength. Several limitations must also be considered, including the limited number of mouthwash DNA samples in the pediatric age group, the lack of inclusion of fathers for comparisons, and the largely white study population. We also were not able to evaluate more recently developed DNA collection methods, such as Oragene saliva collection kits (DNA Genotek, Ontario, CA) that have been shown to yield suitable quantities of DNA in children [23].

## Conclusions

As the focus of epidemiology studies becomes increasingly molecular, collection of DNA samples using cost-effective, reliable, and non-invasive methods is important. The low yields observed in most children in these studies indicate that buccal brush collection is not an ideal method for DNA collection in small children and that development of alternative methods is warranted.

## Methods

### Infant leukemia study

Detailed information regarding case and control ascertainment for the infant leukemia study has been described [24]. Briefly, cases were eligible for the study if they were diagnosed with acute lymphoblastic leukemia or acute myeloid leukemia prior to one year of age at a participating COG institution, did not have a diagnosis of Down syndrome, and had an English or Spanish speaking mother who was available for a telephone interview. Cases were recruited in two phases: 1) January 1, 1996 – October 13, 2002 and 2) January 1, 2003 – December 31, 2006.

Controls were also recruited in two phases corresponding to the time period for case ascertainment. Controls in Phase 1 were recruited using random digit dialing (RDD) using a modification of the methods of Waksberg [25]. Controls in Phase 2 were recruited through state birth registries from 15 states that recruited 62% of eligible cases in Phase 1. Controls were frequency matched to cases based on year of birth and region of residence and were required to have an English or Spanish speaking mother available for interview.

Data collection included maternal interview to collect demographic information, exposure history during pregnancy, and family history data. Demographic characteristics used in this analysis include maternal age at DNA collection (< 30 years, 30 – 35 years, 35 – 40 years, > 40 years), maternal education (< high school, high school graduate, some college, college degree, advanced degree), race (white, other), and household income (< $20,000, $20,000 – $50,000, $50,000 – $75,000, and > $75,000).

This study was approved by the institutional review board at the University of Minnesota. Informed consent was obtained from all participants.

### Hepatoblastoma study

Cases and controls were recruited for the HB study as previously described [26]. Briefly, cases were eligible for the study if they were diagnosed with HB at a COG institution between January 2000 and December 2008 at age ≤ 6 years. Additional eligibility criteria included birth in the United States and having an English- or Spanish-speaking birth mother available for interview.

Controls were recruited through rosters of randomly selected births provided by 32 state birth registries as described [27]. Controls were eligible for the study if they were born in the United States between 1994–2008 and if they had an English- or Spanish-speaking birth mother available for a telephone interview. Controls were frequency matched to cases on birthweight category (< 1500, 1500–2500, and > 2500 g), sex, year of birth and geographic region of birth.

Data were collected from birth mothers of cases and controls by a standardized computer-assisted telephone interview. The interview included information on demographics, pregnancy history, maternal exposures, and family history of cancer. For this analysis, we evaluated the demographic variables listed above.

This study was approved by the institutional review board at the University of Minnesota and each participating COG institution. The study was also reviewed and approved by the state health departments that provided birth certificate data. Informed consent was obtained from all participants.

### DNA collection

Buccal cell DNA was collected for mothers and children using mouthwash and cytobrush collection kits, respectively. Two different types of cytobrushes were used for collection from children in both studies, including Epicentre Catch-All Sample Collection Swabs (QEC091H, Epicentre Biotechnologies, Madison, WI) and Cyto-Pak Cytosoft Brushes (Cat. # CP-5B, Medical Packaging Corporation, Camarillo, CA). For the child’s sample, mothers were instructed to firmly brush the inside of the child’s cheek approximately 20 times with the swab and then to return the swab to the plastic container. The process was repeated on the other cheek with a separate swab.

Mouthwash collection kits were used to collect DNA from case and control mothers in both studies and a small subset of the older children in the HB study. Participants were mailed a small bottle of Scope mouthwash (Proctor and Gamble, Cincinnati, OH) and a sample collection jar. They were instructed to swish Scope vigorously in their mouth for 30–60 seconds prior to spitting into the sample collection container. This process was repeated until the Scope container was empty. The samples were then placed in a sealable plastic bag. All DNA samples were returned through the mail in a prepaid mailer.

### DNA storage and extraction

Upon receipt in the laboratory, buccal brush DNA was stored at 4°C until DNA isolation. Mouthwash samples were stored at −20°C until DNA isolation. DNA was extracted from buccal brushes, swabs, and mouthwash samples using the Puregene Buccal Cell DNA Isolation protocol (Gentra Systems, Minneapolis, MN) according to the manufacturer’s protocol.

### DNA quantification

DNA was quantified using Quantitative Real-Time PCR (qPCR). A standard curve from 0.01 ng to 1000 ng was generated using human genomic DNA (Promega Corporation, Madison, WI). The standards and extracted DNA were assayed in triplicate in a 96-well plate. The extracted DNA was diluted 10-fold before assaying. ABI Taqman RNase P Detection Reagent labeled with FAM and ABI Taqman Gene Expression Master Mix (Applied Biosystems Inc, Foster City, CA) were used to perform the assay. The plate was analyzed using the ABI PRISM 7900HT Sequence Detection System (Applied Biosystems Inc.). Results were accepted when the R^2^ value was greater than or equal to 98% with greater than 90% PCR efficiency and do not exceed a cycle threshold (Ct) standard deviation of 0.25.

### Statistical analysis

All statistical analyses were conducted in SAS v.9.2 (SAS Institute, Cary, NC). The outcome variable (DNA yield) did not follow a normal distribution and thus was log_e transformed for all analyses. Following the transformation, the distribution was approximately normal. A two sample *t*-test with unequal variance was used to compare the yield of DNA in mothers and children in the two studies. Correlations between DNA yield in mothers and their children were evaluated using the Pearson correlation coefficient. Univariate and multivariable linear regression models were used to identify predictors of DNA yield. Variables that were included as potential predictors of DNA yield include: age (mother, child) at DNA collection, sex (for children), case–control status of child, buccal cell collection method (brush type or mouthwash), maternal education, maternal race, household income, DNA storage time in the laboratory, and season of DNA collection (Winter: DJF, Spring: MAM, Summer: JJA, Fall: SON). Analyses were conducted separately for children and mothers. Initial analyses were stratified by study population (HB or infant leukemia). Combined analyses of the two studies were also conducted with adjustment for study population. All reported *P* values are two-sided.

## Competing interests

The authors have no competing interests to disclose.

## Authors’ contributions

JNP, JAR, and LGS were responsible for conception, design, and implementation of the study. AJH, EL and CB conducted all laboratory assays for the manuscript. JNP was responsible for statistical analysis and writing the manuscript. All authors were responsible for reviewing and editing the manuscript. All authors have approved of the final submitted manuscript.
